# FB-CCNN: A Filter Bank Complex Spectrum Convolutional Neural Network with Artificial Gradient Descent Optimization

**DOI:** 10.3390/brainsci13050780

**Published:** 2023-05-10

**Authors:** Dongcen Xu, Fengzhen Tang, Yiping Li, Qifeng Zhang, Xisheng Feng

**Affiliations:** 1 State Key Laboratory of Robotics, Shenyang Institute of Automation, Chinese Academy of Sciences, Shenyang 110016, China; xudongcen@sia.cn (D.X.); lyp@sia.cn (Y.L.); zqf@sia.cn (Q.Z.); fxs@sia.cn (X.F.); 2 Institutes for Robotics and Intelligent Manufacturing, Chinese Academy of Sciences, Shenyang 110169, China; 3 University of Chinese Academy of Sciences, Beijing 100049, China

**Keywords:** deep learning, FB-CCNN, CNN, filter bank, SSVEP, BCI

## Abstract

The brain–computer interface (BCI) provides direct communication between human brains and machines, including robots, drones and wheelchairs, without the involvement of peripheral systems. BCI based on electroencephalography (EEG) has been applied in many fields, including aiding people with physical disabilities, rehabilitation, education and entertainment. Among the different EEG-based BCI paradigms, steady-state visual evoked potential (SSVEP)-based BCIs are known for their lower training requirements, high classification accuracy and high information transfer rate (ITR). In this article, a filter bank complex spectrum convolutional neural network (FB-CCNN) was proposed, and it achieved leading classification accuracies of 94.85 ± 6.18% and 80.58 ± 14.43%, respectively, on two open SSVEP datasets. An optimization algorithm named artificial gradient descent (AGD) was also proposed to generate and optimize the hyperparameters of the FB-CCNN. AGD also revealed correlations between different hyperparameters and their corresponding performances. It was experimentally demonstrated that FB-CCNN performed better when the hyperparameters were fixed values rather than channel number-based. In conclusion, a deep learning model named FB-CCNN and a hyperparameter-optimizing algorithm named AGD were proposed and demonstrated to be effective in classifying SSVEP through experiments. The hyperparameter design process and analysis were carried out using AGD, and advice on choosing hyperparameters for deep learning models in classifying SSVEP was provided.

## 1. Introduction

A brain–computer interface (BCI) provides direct communication between human brains and machines without using peripheral nerves or muscles [1], thus allowing users to use brain signals to control devices such as spelling interfaces [2,3], wheelchairs [4,5], robot arms [6,7], drones [8,9], exoskeletons [10,11] and robots [12,13]. Among the different BCIs, BCIs based on electroencephalography (EEG) are the most widely used due to their convenience, safety, low cost and high temporal resolution [14]. There are multiple commonly-used physiological EEG paradigms, including P300 [15], motor imagery [16] and steady-state visual evoked potential (SSVEP) [17]. Of these three paradigms, SSVEP has the advantages of requiring less training, a high information transfer rate and high accuracy. SSVEP is an oscillatory electrical potential that is generated in the brain when subjects are watching stimuli flickering at a frequency of 6 Hz or higher [17]. SSVEPs arise from a reorganization of spontaneous intrinsic brain oscillations in the presence of a stimulus [18] most evident in the occipital region. SSVEPs have the same fundamental frequency as the stimulus and its harmonics [19].

There are five main processing stages in a BCI: a data collection stage that records neural data from the brain, a signal processing stage that cleans the noise from the data, a feature extraction stage that generates and amplifies features to make them easier to classify, a classification stage that produces the output of the BCI using the features from the last stage and a feedback stage that presents the output of the BCI to the subject [20]. The core of a BCI is the classification stage, which determines the performance of the BCI given the same neural data. Machine learning technology is widely used at the classification stage [21] of BCI, yet with the advancement of deep learning technology, an increasing number of researchers have started to apply deep learning to the classification of BCIs, including SSVEP-based BCIs [22,23,24].

Although deep learning is a powerful tool for classifying complex data, the performance of a deep learning model largely depends on its structure and the size of the training data; without enough data to train the deep learning model’s weights, the more complex the deep learning model is, and the worse it will perform [25]. Unlike computer vision which has millions of pictures for deep learning models to train on, the amount of SSVEP data is limited in quantity and may not be enough to support the training of highly complex deep learning models. Most of the deep learning models for classifying SSVEP implement a convolutional neural network (CNN), as CNNs take advantage of the local spatial coherence of SSVEP signals either in the time domain or in the frequency domain so that the CNN model has fewer weights and is easier to train [22].

Kwak et al. built three deep learning models for classifying SSVEPs, two CNN models with two and three hidden layers, respectively, and one artificial neural network (ANN) model that is fully connected. Kwak et al. found that the CNN model with fewer hidden layers and a lower complexity performed better [26]. However, a simpler CNN model is not always better. Aznan et al. found that a CNN model with one convolutional layer worked well for one subject, but when the model was applied to another subject, the performance of the model dropped significantly compared to a CNN model with five convolutional layers, which suggests that a more complex CNN model may have a better generalization ability [27]. Zhao et al. built a CNN model with five hidden layers to classify AR-SSVEP signals. Zhao et al. tested the performance of ensemble-TRCA, CCA and FBCCA and their CNN model and found that their CNN model had significantly better performance [28]. A CNN model’s performance is sensitive to the complexity of the input data, and it will decrease when the complexity of the input surpasses a certain point. Podmore et al. built a CNN model to classify SSVEPs; when the input was three-channel SSVEP data, their model performed better than FBCCA, but when the input was five-channel SSVEP data, their model performed worse than FBCCA [29].

To enhance the performance of CNN models in classifying SSVEPs, many researchers use fast Fourier transform (FFT) to make features of SSVEP data easier to be extracted by the CNN models. Kwak et al. used FFT to transform time domain data to 120 frequency samples before feeding them to the CNN model [26]. Nguyen et al. applied FFT to single-channel data to amplify the features in the input data for SSVEP classification [30]. Dang et al. used the FFT of the input to intercept the fundamental wave spectrum with its harmonics and concatenated them together as input to a CNN model [31]. The FFT output of the SSVEP data has real parts and imaginary parts, magnitude information and phase information. Ravi et al. found that, in their CNN model, using its complex spectrum by concatenating the real parts and complex parts of the FFT output together as the CNN model’s input had a higher classification accuracy compared to using the magnitude spectrum of the FFT data [32].

Another commonly used preprocessing technique is filter banks. In 2015, Chen et al. found that by adding filter banks to the traditional classification method canonical correlation analysis (CCA), the new method performed significantly better than CCA by improving the average accuracy from 76.8% to 91.95% [33]. Filter banks are also used in CNN models to improve their performances. In 2021, Ding et al. compared two CNN models’ performances using time domain SSVEP data as the input, one set with filter banks and one without. Ding et al. found that by adding filter banks to the preprocessing of model input, the FB-tCNN model’s performance had a 5.53% increase in accuracy using their own dataset and a 5.95% increase using a public dataset [34]. In the same year, Zhao et al. built an FB-CNN model that implements three filter banks and a CNN model with three convolutional layers before concatenating them together into a fully connected layer. Compared to a C-CNN model that does not implement a filter bank technique, the FB-CNN had better accuracy using two open datasets [35]. In 2022, Pan et al. added four filter banks to process the input of their CNN model and outperformed other traditional or deep learning benchmark methods in classifying SSVEPs [36]. Additionally, in 2022, Chen et al. incorporated filter banks into their transformer-based model and found that the best performance of the model was obtained when using three filter banks, compared to using two or four filter banks [37]. In 2022, Yao et al. built three filter banks to preprocess SSVEPs and fed them separately into three individual EEGNet models before merging the extracted features together into a fully connected layer. The model outperformed EEGNet [38]. Bassi et al. built three deep-learning models with filter banks to classify SSVEPs in 2022. Of the three models, one of them was a FB-RNN, and the other two were FB-CNNs in 2D and 3D, respectively. Bassi et al. utilized 10 filter banks to preprocess the SSVEP data [39]. Filter banks have become a powerful tool in preprocessing SSVEP data and are widely used with deep learning models to boost the deep learning models’ performances.

However, although there are many CNN models that achieve high accuracies in classifying SSVEPs, very few of them describe the process of choosing hyperparameters for the CNN models, including the size of kernels, the number of kernels and the stride of the convolutional layers [22]. Although the design of CNN models is more like a trial-and-error process, the process of optimizing the model to its best performance is important [25].

In this paper, an FB-CCNN is proposed to classify SSVEPs, and an algorithm for generating and optimizing the hyperparameters of deep learning models, including FB-CCNN, is proposed. The FB-CCNN implements filter banks to preprocess SSVEPs, then uses FFT to transfer the time domain data into frequency domain data, with real parts and complex parts of the complex spectrum data concatenated together as input to the CNN model. After the initial design of FB-CCNN, AGD was used to choose the hyperparameters of the model. There are four main contributions of this paper:

- A novel deep learning model named FB-CCNN is proposed and validated to have leading classification performances using two open SSVEP datasets.
- A hyperparameter optimization algorithm named artificial gradient descent is proposed and validated to be effective in choosing deep learning model’s hyperparameters.
- A hyperparameter relationship analysis was carried out by training 243 models using AGD; this is the first hyperparameter analysis used in SSVEP deep learning models, and it revealed the correlations between hyperparameters and the model’s performance.
- Through experiments, it was demonstrated that the hyperparameters of the FB-CCNN should be fixed values rather than channel number-based, as in most CNN models, and using filter banks allows the model to have a better generalization ability.
- In Section 2, two SSVEP open datasets and benchmark methods for comparison purposes are introduced. FB-CCNN and AGD are presented and used to generate four sets of hyperparameters. In Section 3, FB-CCNN with four sets of hyperparameters are tested on two open datasets, and the results are shown. Section 4 is a discussion of the experimental results, limitations and future directions. The conclusion is provided in Section 5.

## 2. Materials and Methods

This section introduces the two SSVEP open datasets used in this study. Then, the benchmark methods for comparison purposes are presented. FB-CCNN and AGD are explained in theory and demonstrated using experiments. Four sets of FB-CCNN hyperparameters were generated by AGD and used for testing in the next section.

### 2.1. Datasets

In this work, two SSVEP open datasets were employed to evaluate the performance of our proposed FB-CCNN method. The first open dataset was generated by Nakanishi in 2015, named the Nakanishi dataset [40], and the second open dataset was generated by Wang in 2016, named the Benchmark dataset [41].

#### 2.1.1. Nakanishi Dataset

The Nakanishi dataset contains SSVEP data from 10 healthy subjects with normal or corrected-to-normal vision. They were presented with 12-target visual stimuli, flickering with different frequencies ($f_{0}=9.25$ Hz, Δf = 0.5 Hz) and phases (${Ø}_{0}=0$, ΔØ = 0.5π). The SSVEP data were collected using eight electrodes at a sampling rate of 2048 Hz. For each subject, the experiment consisted of 15 blocks, and in each block, the subjects were asked to gaze at one of the visual stimuli indicated by the stimulus program in a random order for 4 s; the subjects completed 12 trials corresponding to all 12 targets. A red square appeared for 1 s to guide the subjects to shift their gaze before the stimuli flickered for 4 s on the monitor. All data epochs were later downsampled to 256 Hz and then band-passed and filtered from 6 Hz to 80 Hz using an infinite impulse response (IIR) filter. As there is a latency delay in the visual system, all data epochs were extracted with a 0.135 s delay after the stimulus onset [40]. The Nakanishi dataset can be obtained at: https://github.com/mnakanishi/12JFPM_SSVEP (accessed on 31 March 2023).

#### 2.1.2. Benchmark Dataset

The Benchmark dataset contained SSVEP data from 35 healthy subjects with normal or corrected-to-normal vision. Eight of them had experience using SSVEP BCIs, and twenty-seven of them had no experience using an SSVEP BCI. The subjects were presented with 40-target visual stimuli, flickering with different frequencies ($f_{0}=8$ Hz, Δf = 0.2 Hz) and phases (${Ø}_{0}=0$, ΔØ=0.5π). The SSVEP data were collected using 64 electrodes at a sampling rate of 1000 Hz. For each subject, the experiment consisted of six blocks, and in each block, the subjects were asked to gaze at one of the visual stimuli indicated by the stimulus program in a random order for 5 s; the subjects completed 40 trials corresponding to all 40 targets. A red square appeared for 0.5 s to guide the subjects to shift their gaze before the stimulus concurrently flickered for 5 s on the screen. All data epochs were later downsampled to 250 Hz to reduce storage and computation costs. No digital filters were applied in the data preprocessing [41]. The Benchmark dataset can be obtained at: http://bci.med.tsinghua.edu.cn/download.html (accessed on 31 March 2023).

### 2.2. Benchmark Algorithms

CCA, C-CNN [32] and FB-CNN [35] were chosen as benchmark methods for comparison.

#### 2.2.1. Canonical Correlation Analysis

CCA is a traditional machine-learning method for classifying SSVEPs and is widely used in SSVEP-based BCIs [42]. Given SSVEP data *X* ∈ $R^{N_{x}ⅹN_{s}}$ and template signal *Y* ∈ $R^{N_{y}ⅹN_{s}}$, CCA finds two spatial filters, $w_{x}$ ∈ $R^{N_{x}ⅹ1}$ and $w_{y}$ ∈ $R^{N_{x}ⅹ1},$ which maximize the correlation between *x* = $X^{T}w_{x}$ and *y* = $Y^{T}w_{y}$ by solving the following optimization problem,

(1) $$\rho \left(X,Y\right)=\underset{w_{x},w_{y}}{\max}\frac{E\left[w_{x}{}^{T}XY^{T}w_{y}\right]}{\sqrt{E\left[w_{x}{}^{T}XX^{T}w_{y}]E[w_{y}{}^{T}YY^{T}w_{y}\right]}}$$

where ρ(X,Y) is *X* and *Y*’s maximal canonical correlation.

The template signal *Y* is composed of a series of sine and cosine waves, which have frequencies corresponding to the stimuli frequencies and their harmonics. The output of the classification by CCA is the frequency which produces the maximum correlation between X and Y, and is considered the stimulus frequency that the subject is staring at.

#### 2.2.2. Complex Convolutional Neural Network

C-CNN was proposed by Ravi et al. in 2020. It is a CNN model with two hidden layers and uses a complex spectrum of FFT data as input. It has two convolutional layers, each with 2 × $N_{ch}$ kernels, and their corresponding kernel sizes are $N_{ch}\times 1$ and 1 × ($N_{FFT}-9$). C-CNN was demonstrated to outperform CCA, FBCCA, TRCA and M-CNN, a CCN model with the same structure as C-CNN but uses the magnitude spectrum of FFT data as input [32]. $N_{ch}$ is the channel number of the SSVEP data, and $N_{FFT}$ is the number of frequency components after FFT.

#### 2.2.3. Filter Bank Convolutional Neural Network by Zhao

In 2021, Zhao et al. built a FB-CNN model that implemented three filter banks as a preprocessing module. The complex spectrum of FFT data from the three filter banks was fed separately into three individual CNN subnets that each had three convolutional layers; then, the output of the CNN models was flattened and fully connected to the output layer. The three filters had passbands of 6–16 Hz, 16–32 Hz and 32–64 Hz, respectively. The first two EEG channels were repeated in the input layer to allow for the “valid” padding mode. The first convolutional layer implemented a 3 × 3 kernel to extract the input layer features, and the kernel sizes of the second and third convolutional layers were $N_{ch}\times 1$ and 1 × ($N_{FFT}-2$), respectively [35].

### 2.3. The FB-CCNN Model

**Figure 1:**
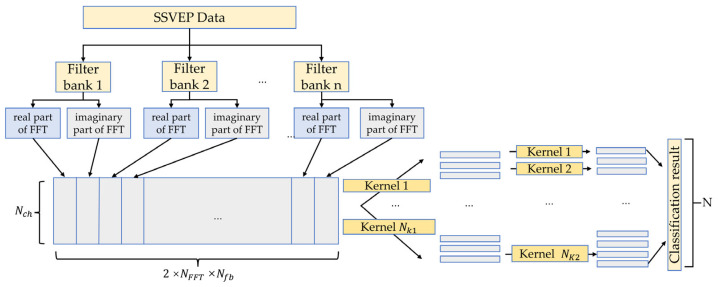
The architecture of filter bank complex spectrum convolutional neural network (FB-CCNN). $N_{ch}$ represents the number of channels of SSVEP data, $N_{FFT}$ represents number of frequency components after FFT, $N_{fb}$ represents number of filter banks, $N_{k1}$ is the number of kernels in the first convolutional layer, and $N_{k2}$ is the number of kernels in the second convolutional layer.

Filter banks have been demonstrated in many works to significantly improve performance in classifying SSVEPs when used with machine learning models or deep learning models [33,35,37,38]. C-CNN demonstrated that using a complex spectrum of FFT data was more effective than using the magnitude spectrum of FFT data and performed well in classifying SSVEPs. In this paper, the FB-CCNN model proposed has two main components: one is a filter bank that preprocesses SSVEP data using different filters, and the other is a convolutional neural network that uses a complex spectrum of FFT data as input, as shown in Figure 1.

#### 2.3.1. The Filter Bank Component

The filter bank component is composed of $N_{fb}$ filters, each with a different passband of zero-phase Chebyshev Type I Infinite Impulse Response (IIR) filters. The filter bank is used to decompose the SSVEP data into different sub-band components so that the harmonic information of the SSVEP data can be analyzed independently and then grouped together for higher classification accuracy.

For most SSVEP BCIs, including the two open datasets used in this paper [40,41], the bandwidth of stimulation was less than or equal to 8 Hz. According to Chen’s research [33], filter banks with passbands starting from *n* × 8 Hz and ending at 88 Hz perform the best, where *n* ∈ [1, 2, …, $N_{fb}$]. FB-CCNN follows the same design as Chen for the filter banks. However, the optimal $N_{fb}$ varies between different studies. In Chen’s FBCCA algorithm, experiments determined the best $N_{fb}$ as 7, but in other deep-learning studies that implemented the filter bank technique, the values of $N_{fb}$ were different. In Yao’s FB-EEGNet [38] and Zhao’s FB-CNN model [35], $N_{fb}$ was chosen to be 3. In Ding’s FB-tCNN, $N_{fb}$ was chosen to be 4 for an open dataset and 3 for his own dataset [34]. In Bassi’s FB-DNN model, $N_{fb}$ was chosen to be 10 [39]. In the deep learning studies mentioned above, the selection of $N_{fb}$ values was not validated by experiment or theory. In this paper, the model performance using different $N_{fb}$ was compared, including when $N_{fb}$ = 1, which means one filter bank is used, which in this case, is the same as using a single filter.

The filter bank component filters the SSVEP data with different passbands and then concatenates them together into a 2D matrix of size $N_{ch}$ × (2 × $N_{FFT}\times N_{fb}$), where $N_{ch}$ is the channel number of the SSVEP data and $N_{FFT}$ is the number of frequency components extracted by FFT. For one filter, after FFT, it will produce a matrix of $N_{ch}\times N_{FFT},$ representing the real part of the FFT data, and a matrix of $N_{ch}\times N_{FFT},$ representing the complex part of the FFT data concatenated together, producing a matrix of size $N_{ch}$ × (2 × $N_{FFT}$). With $N_{fb}$ filter banks, the output matrix size is $N_{ch}$ × (2 × $N_{FFT}\times N_{fb}$), as shown in Figure 1.

#### 2.3.2. The Complex Spectrum Convolutional Neural Network Component

The complex spectrum convolutional neural network is composed of two convolutional neural layers and one fully connected layer. The first convolutional layer utilizes a kernel of size [$N_{ch}$ × 1] and stride of 1 to extract the FFT features from all of the channels and merge them together as a new feature for the next convolutional layer to work on. Dropout is implemented to prevent overfitting with a dropout rate of 0.5, and batch normalization is implemented to make the training faster and more stable [25].

The number of kernels of convolutional layer 1 is represented by $N_{k1}$. In Ravi et al.’s C-CNN model and Zhao et al.’s FB-CNN model, $N_{k1}$ = 2 × $N_{ch}\;$[32,35]. In Bassi’s FB-CNN 2D model, $N_{k1}$ = 16. In the first two works, the optimal kernel number of the first layer is represented as a multiple of the channel number, while in the last work, the optimal kernel number is a fixed number (16). These selections were not validated to be the optimal selection in their articles; in this article, $N_{k1}$ was determined using artificial gradient descent (AGD).

The rest of the hyperparameters were chosen in the same way using AGD, including the number of kernels in the second convolutional layer $N_{k2}$, which has a size of 1 × $K_{2},$ while $K_{2}$ is also a hyperparameter. The second convolutional layer has a stride of $S_{2}$ which is another hyperparameter. The second convolutional layer also implements dropout with a dropout rate of 0.5 to prevent overfitting as well as batch normalization.

The last layer is a fully connected layer that connects every element in previous convolutional layers to the N neurons which represents N targets. The last fully connected layer has $N_{f}$ × *N* connections as follows:

(2) $$N_{f}=(\lfloor \frac{2\times N_{FFT}\times N_{fb}-K_{2}}{S_{2}}\rfloor +1)\times N_{k2}$$

All of the hyperparameters, including $N_{fb}$, $N_{k1}$, $N_{k2}$, $K_{2}$ and $S_{2},$ in the FB-CCNN were generated and optimized by artificial gradient descent.

For the activation function, C-CNN, FB-CNN and 2D FB-CNN all adopt ReLU as the activation function. However, in 2010, Nair and Hinton showed that ReLU activation has a significant limitation in that it is sometimes fragile when the deep learning model is being trained and sometimes causes the gradients to die, leading to dead neurons and thereby stopping the updating of weights during training and hindering the learning process as dead neurons give zero activation [43]. Parametric ReLU (PReLU) was proposed by He in 2015 and was considered to be better than ReLU in large-scale image training, and this model that implemented PReLU was the first deep learning model to surpass human-level performance on a visual recognition challenge [44]. Therefore, to offer better performances in SSVEP recognition, FB-CCNN implements PReLU as the activation function in all layers.

### 2.4. Artificial Gradient Descent (AGD)

**Figure 2:**
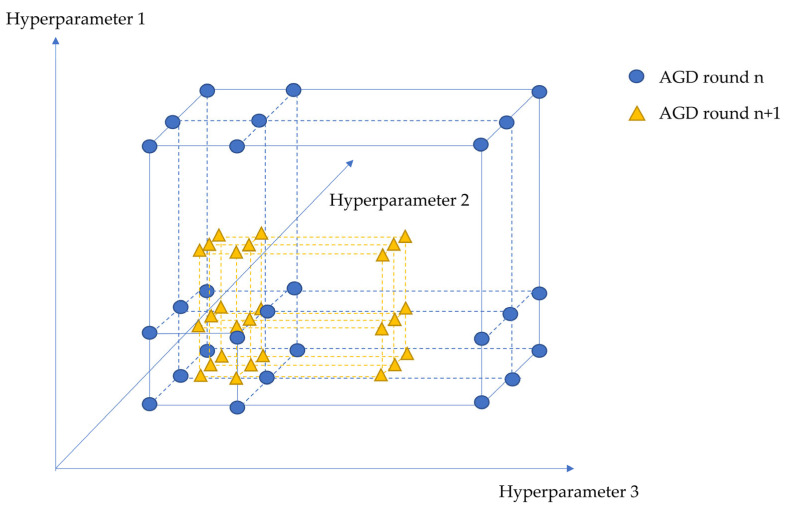
A demonstration of the AGD searching process with 3 hyperparameters at round *n* and *n* + 1.

Artificial gradient descent (AGD) is an artificial hyperparameter searching method that works very similarly to gradient descent. In gradient descent, the parameters of a deep learning model are optimized in each run to generate a smaller loss of the model. In artificial gradient descent, researchers need to manually select the deep learning model’s hyperparameter set according to the performance of the model in each run of AGD, including the initial hyperparameter values that need to be optimized. The algorithm of artificial gradient descent for hyperparameter searching is shown in Table 1. To better illustrate the principle of AGD, a 3-hyperparameter searching process using AGD in round *n* and *n* + 1 is demonstrated in Figure 2.

The AGD algorithm was designed to generate the FB-CCNN’s hyperparameters, but it can also be applied to other deep-learning models. The manual selection in each run saves computation power and time, and the number of selected sets of hyperparameters depends on the computation power and computation time allowed to develop the deep learning models.

To generate the hyperparameters for the FB-CCNN, in the first run of AGD, the following hyperparameter sets were chosen (Table 2) according to previous studies, which is better than random starting values. Additionally, they were rounded to the nearest integer as these hyperparameters need to be integers.

For $N_{fb}$, the value was not chosen according to the AGD initialization ratio because 3 was the most frequently used sub-bands number, and 7 was validated to be the optimal sub-band number for FBCCA. To conduct the first round of artificial gradient descent, $3^{5}$ = 243 models were generated and tested, which is a large number in training a comparatively complex deep learning model.

Subject S2′s SSVEP data from the Nakanishi dataset were used to generate the hyperparameters to save computation power instead of using all of the subjects’ data, which is recommended in the case of sufficient computation power [40]. Subject S2 was chosen as S2 had the lowest classification accuracy in CCA and C-CNN, which can provide a higher difference in testing accuracies and facilitate the searching process for the best-performing hyperparameter set. Of the 15 trials of subject S2, the data from 12 trials were selected for training, and the data from 3 trials were selected for testing the performance of the model. The data length used for one classification was 1 s and was segmented using a space of 0.1 s to generate more training data and testing data. The frequency resolution of FFT was 0.293. The number of total epochs of the training batches was 50. The Adam optimization method and batch training with a batch size of 32 was used for training [45].

**Figure 3:**
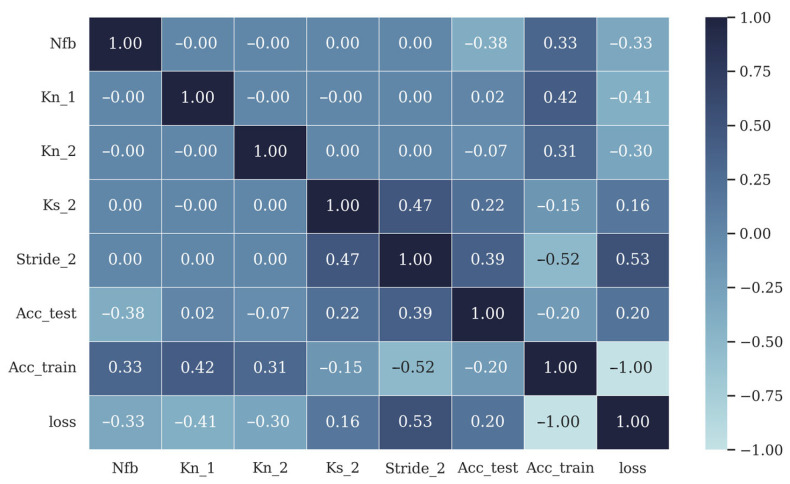
Correlation analysis for hyperparameters’ impacts on model performance using the performance of the 243 models after AGD. $N_{fb},$ represents N_fb, Kn_1 represents $N_{k1}$, Kn_2 represents $N_{k2}$, ks_2 represents $K_{2}$, Stride_2 represents $S_{2}$, Acc_test represents test accuracy, Acc_train represents training accuracy and loss represents the model loss in training.

After the training of the 243 models using Google Colab Pro+, which is the first round of AGD, a correlation analysis was performed to analyze the relationship between test accuracy and the hyperparameters, as shown in Figure 3. From Figure 3, the following conclusions can be made:

- The filter bank number $N_{fb}$ had a strong negative impact on the test accuracy, and thus should not be too high and less than 7.
- The kernel size $K_{2}$ in the second convolutional layer and the stride $S_{2}$ impacted the test accuracy much more than the kernel numbers $N_{k1}$ and $N_{k2}$ in the first and second convolution layers.

**Figure 4:**
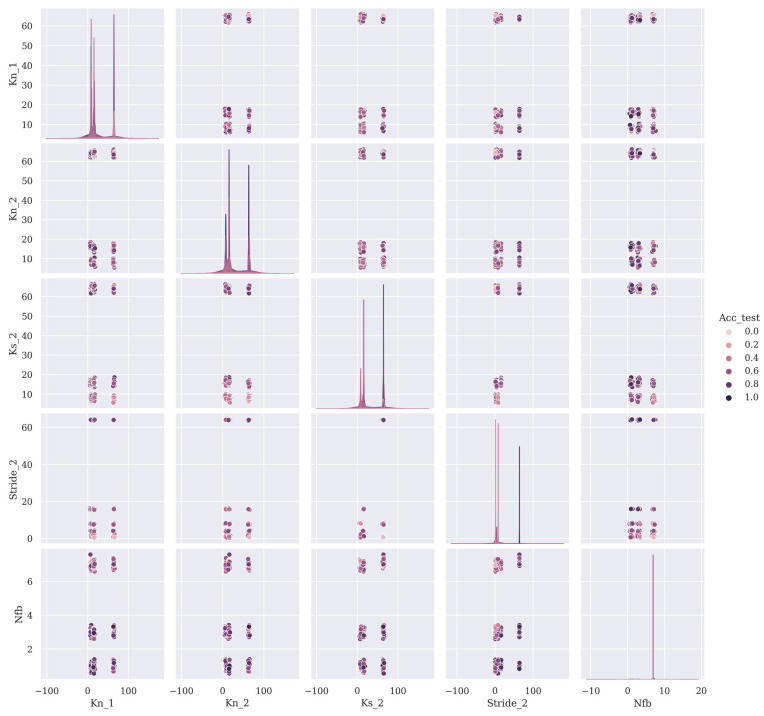
A pairwise analysis for test accuracy using first-round AGD results from subject S2′s SSVEP data in the Nakanishi dataset. The abbreviations in Figure 4 are the same as those in Figure 3.

The relationships between the values of the hyperparameters and the test accuracy of subject 2 using the performance of the 243 training models after AGD round 1 are shown in Figure 4 using a pairwise analysis. Here, to amplify the differences in Figure 4, the test accuracy was normalized to 0–1. From Figure 4, the following conclusions can be made:

- The model performed better when the values of $K_{2}$ and $S_{2}$ were higher.
- Generally, the model did not perform well when $N_{fb}$ was 7 and, therefore, $N_{fb}$ should be smaller.
- The model had a similar performance when $N_{fb}$ was 1 or 3.

**Figure 5:**
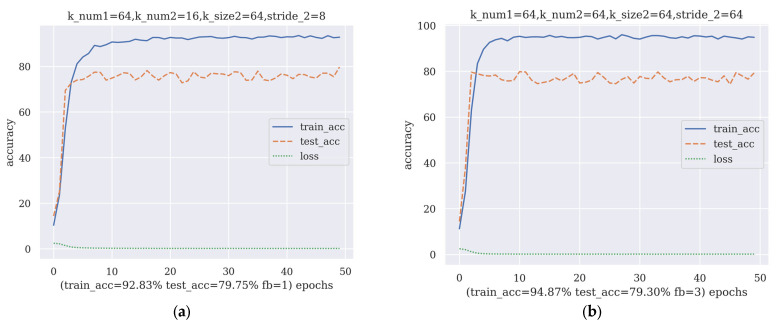
The manually chosen hyperparameter sets: (**a**) for set_ A, k_num1 is $N_{k1}$, k_num2 is $N_{k2}$, k_size2 is $K_{2}$, stride_2 is $S_{2}$, fb is $N_{fb}$, train_acc is the training accuracy at the 50th epoch and test_acc is the test accuracy at the 50th epoch; (**b**) for set_B, the abbreviations have the same meaning as those for set_A.

After the first round of AGD, the average training accuracy and average test accuracy were calculated. The average training accuracy was 89.11%, and the average test accuracy was 72.4%. Then, two sets of hyperparameters, set_A and set_B, with high training accuracy, high test accuracy and low loss, were chosen as the starting sets for the next round of AGD. set_A and set_B’s model performances are shown in Figure 5. Note that set_A uses one filter bank, which means it uses a single filter to preprocess the input SSVEP data.

As Figure 3 shows, the number of kernels in convolutional layer 1 $\left(N_{k1}\right)$ and the number of kernels in convolutional layer 2 ($N_{k2}$) had no significant relationship with test accuracy, and therefore, the second round of AGD did not include an update of these two values. For $N_{fb}$, two values of $N_{fb}$, 1 and 3, were chosen to examine the effectiveness of filter banks in FB-CCNN. The hyperparameter sets optimized from set_A and set_B had values determined using AGD and are shown in Table 3.

**Figure 6:**
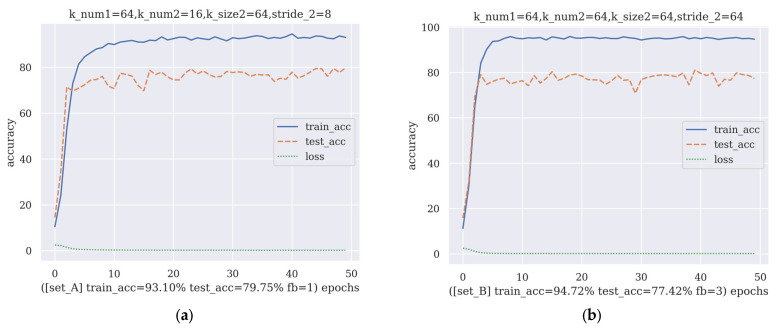
The two hyperparameter sets given by the second round of AGD which offer the best performance: (**a**) hyperparameter set when filter bank number = 1 and (**b**) hyperparameter set when filter bank number = 3. The definitions of the abbreviations in Figure 6 are the same as those in Figure 5.

Note that there is an upper bound for $K_{2}$; $K_{2}$ will not be larger than $2\times N_{FFT}\times N_{fb},$ which is the number of neurons of the previous convolutional layer’s output. Additionally, in set_B, when the stride is larger than the size of the kernel, it indicates that some output of the previous convolution layer was neglected. Therefore, the maximum value of $S_{2}$ is $K_{2}.$ Additionally, the reason this algorithm is named artificial gradient descent is that, in the search for hyperparameter values, sometimes the range of searching will go beyond reasonable limits; thus, it needs an artificial adjustment to avoid a waste of computation power and facilitate the training process. The best-performing hyperparameter sets in set_A and set_B after the second round of AGD are shown in Figure 6.

By comparing Figure 5 and Figure 6, the improvement in test accuracy during the second round of AGD is trivial, so AGD ends at round 2 for the FB-CCNN. Additionally, the hyperparameter sets obtained after two rounds of AGD are shown in Table 4.

For the Nakanishi dataset, $N_{ch}$ = 8. However, for the Benchmark dataset, $N_{ch}$ = 64, which is significantly higher than the $N_{ch}$ of the Nakanishi dataset. Although in previous studies, the hyperparameters of the CNN model depend on the number of channels used in the SSVEP data; whether this approach works better than the fixed value approach has not been validated. The next section describes the experiments that were conducted to determine whether channel number-based hyperparameters or fixed value hyperparameters perform better and to validate and compare the performance of FB-CCNN with those of other benchmark methods.

## 3. Results

To test the performance of our model, FB-CCNNs with a channel number-based hyperparameter set or fixed value hyperparameter set were tested using two open datasets, the Nakanishi dataset and the Benchmark dataset, as shown in Table 5. The fixed value hyperparameters and channel number-based hyperparameters were the same for the Nakanishi dataset because the fixed value hyperparameters were obtained by putting the value of the channel number into the channel number-based hyperparameters of the Nakanishi dataset. However, as the Benchmark dataset has different channel numbers, the values of the hyperparameters differ in these two cases.

### 3.1. Validation Using Nakanishi Dataset

**Figure 7:**
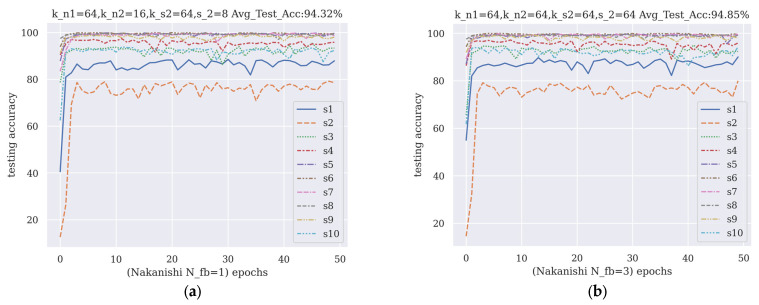
The two hyperparameter sets generated by the second round of AGD: (**a**) hyperparameter set when filter bank number = 1; (**b**) hyperparameter set when filter bank number = 3. The abbreviations in Figure 6 have the same definitions as those in Figure 5.

The performances of the one-filter-bank and three-filter-bank hyperparameter sets using the Nakanishi dataset during training are shown in Figure 7. In the Nakanishi dataset, the channel number-based hyperparameter set was the same as the fixed value hyperparameter set.

The comparison of the performances of the FB-CCNN with other benchmark methods is listed in Table 6. The FB-CCNN had a significantly higher accuracy of classification and stability compared to CCA and C-CNN. The classification accuracy of the FB-CCNN was higher than Fb-CNN, with more stability. Additionally, when applied to the Nakanishi dataset, the difference between using three filter banks and using one filter bank was trivial.

### 3.2. Validation Using Benchmark Dataset

The FB-CCNN was tested using the Benchmark dataset with four sets of hyperparameters. Two of them were fixed-value hyperparameter sets that had the same hyperparameter values as those in the Nakanishi dataset, and the other two were channel number-based hyperparameter sets, as suggested by Ravi and Zhao in their papers [32,35]. The performances of the models using these four hyperparameter sets are shown in Table 7.

From Table 7, it is evident that the FB-CCNN model using a channel number-based hyperparameter set performed significantly worse than the FB-CCNN model that used a fixed value hyperparameter set. The significant increase in the channel number from 8 to 64 made the number of weights in the model increase exponentially, and the training data may become insufficient to train such a large network; thus, the FB-CCNN with fixed hyperparameter values performed better. Additionally, the FB-CCNN with three filter banks performed significantly better than the FB-CCNN with one filter bank, which shows that more filter banks provide the model with better generalization ability. Table 7 shows that the FB-CCNN performed the best among four SSVEP classifying CNN models, and the best performing FB-CCNN had $N_{fb}$ = 3, $N_{k1}$ = 64, $N_{k2}=64,\;K_{2}=64\;and\;S_{2}=64$.

## 4. Discussion

In this section, the experiment results are discussed, together with the limitations of this study and future works.

### 4.1. Structure Design in FB-CCNN

To seek the optimal FB-CCNN structure in classifying SSVEPs, five hyperparameters were optimized using AGD, including filter bank number, kernel size of convolutional layer 2, number of kernels in convolutional layers 1 and 2 and stride of convolutional layer 2. As shown in Figure 3, the number of kernels in convolutional layer 1 was almost irrelevant to the performance of the model, and the number of kernels in convolutional layer 2 had a higher impact than that of convolutional layer 1 but was still significantly less important than the kernel size in convolutional layer 2 and stride of convolutional layer 2. A possible explanation is that the FFT input from the filter banks has strong features; thus, the first convolutional layer does not require many kernels to extract different features from the input. However, the kernels in the second convolutional layer can extract features that cover frequency data from different filter banks in a broad range, and thus more kernels contribute to more diverse interpretations of the features in the data. However, the influence of the number of kernels in the second convolutional layer cannot match the influence of the size of the kernel and stride of the second convolutional layer, as the size of the kernel determines the information scope per kernel, and a larger kernel can extract frequency information from larger frequency intervals. With a larger stride, the number of weights in the convolutional layer drops dramatically, making the CNN network easier to train when there is insufficient training data, and making the CNN network more efficient. For the number of filter banks, from the Nakanishi dataset testing results, it can be observed that using one filter bank had almost the same performance as using three filter banks; however, from the Benchmark dataset testing results, it was obvious that using three filter banks provided the model with a much higher generalization ability, and thus was the optimal choice.

### 4.2. Fixed Value Hyperparameters Performed Better Than Channel Number-Based Hyperparameters

C-CNN and FB-CNN both adopt channel number-based hyperparameters. In C-CNN, the first and second convolutional layers both have 2 × $N_{ch}$ kernels. FB-CNN also has 2 × $N_{ch}$ kernels in its three convolutional layers in three individual CNN models.

The disadvantage of using a channel number-based hyperparameter design is that, when applied to different datasets, the difference between channel numbers is too large for the model to be nearly the same. For example, SSVEP classification can be achieved with just 1 channel or 256 channels, but the difference is too much for the CNN model to have similar performances. As shown in Table 7, the performance of the FB-CCNN dropped significantly when using a channel number-based hyperparameter set, as the number of weights increased dramatically when the number of channels increased to 64 in the Benchmark dataset, but the length of data did not increase proportionally. This leads to a significant increase in the complexity of the model when the dataset is switched from the Nakanishi dataset to the Benchmark dataset without providing more corresponding training data and leads to a decrease in the performance of the model. However, for most datasets, their SSVEP data volume for each subject is close in quantity, which means the training data in different datasets for deep learning models using a fixed value hyperparameter set is likely to be sufficient, as a fixed value hyperparameter set keeps the complexity of the model the same. Additionally, it was shown through experiments that it is better to use a fixed-value hyperparameter set across different SSVEP datasets.

### 4.3. The Value of AGD

The value of AGD is not just in optimizing for the best hyperparameter set to use in a deep learning model; it can also reveal the impact of each hyperparameter on the performance of the model, as shown in Figure 4. The acquisition of the relationship information between the hyperparameters and model performance leads to a more efficient model design process and a more comprehensive understanding of the characteristics of the dataset. In the consecutive rounds of AGD for optimizing the hyperparameters, the trivial hyperparameters can be removed to reduce the number of models to be trained in order to save computation power and time.

### 4.4. Multiple Individual CNN Models or One CNN Model

One of the core differences between the FB-CNN and FB-CCNN is that the FB-CNN uses three individual CNN models to analyze the FFT data from three filter banks separately and then merges their output by flattening and concatenation, while the FB-CCNN uses only one CNN model after concatenating the filter bank output into the input. The result of testing using two open datasets showed that using one CNN model was better than using three individual CNN models and then merging them together. This is probably because when using only one CNN model, there is mutual understanding across features from different filter banks, leading to a better understanding of the input by the model.

### 4.5. Limitation and Future Works

Some limitations of this work should be mentioned. Due to limited computation capacity, the AGD used singular subject data, and thus the performance of the FB-CCNN was optimized using intra-subject classification, and inter-subject performance optimization by AGD is currently unavailable. Additionally, to test the performance of the FB-CCNN and AGD, this study only used open datasets, which is an offline “closed world” scenario. In the future, the following research directions will be followed:

- Testing and optimizing the performance of the FB-CCNN in inter-subject scenarios and comparing it with other benchmark methods.
- Applying the FB-CCNN to an online BCI system and testing its performance with recruited subjects.
- Adding a proportional similarity-based Openmax classifier to the FB-CCNN to detect whether the subject is watching the stimuli on the screen [46] and thus improve the synchronous SSVEP BCI system to an asynchronous SSVEP BCI system to expand its practicality.
- Implement the FB-CCNN for real-time control of devices such as mobile vehicles [47], wheelchairs [48] and robotic arms [49].

## 5. Conclusions

In summary, a novel filter bank complex spectrum convolutional neural network was proposed and demonstrated to have leading classification accuracy and stability compared to previously developed methods. An optimization algorithm named artificial gradient descent was also proposed to optimize the value of the hyperparameters for deep learning models, and its effectiveness was validated in practice. Artificial gradient descent was also demonstrated to be effective in analyzing the relationship and impact between hyperparameters and the deep learning model’s performance. Through experiments, it was demonstrated that, when designing CNN models for SSVEP classification, it is better to use fixed value hyperparameters than to use channel number-based hyperparameters.

## Figures and Tables

**Table 1:** The algorithm of artificial gradient descent (AGD).

Algorithm: Artificial Gradient Descent (AGD)			
	Input: *n* hyperparameter set including: $a_{1}{}^{0}$, $a_{2}{}^{0}$, …, $a_{n}{}^{0}$		
	Output: Optimized *n* hyperparameters		
	Initialization of variables: Assign three values for each of the hyperparameter set that $a_{i}{}^{0}$ ∈ [$k_{i},2\times k_{i},8\times k_{i}$], where *i* ∈ [1, 2, …, *n*], where the range [$k_{i},8\times k_{i}$] covers searching domain for optimal hyperparameter based on experience or estimation		
**L1**	**For each** combination of value in $a_{1}{}^{r},\;a_{2}{}^{r}\ldots a_{n}{}^{r}\;\left(r=0\;\mathrm{initially}\right)$ train the model accordingly and calculate the model’s performance $P_{m}{}^{r}$, where *m* ∈ [1, 2, …, $3^{n}$], and *r* ∈ [0, 1, …, $r^{s}$], here at round $r^{s}$ the model’s performance is good enough		
	**End for**		
	**For each** manually selected optimal performance $P_{m}{}^{r}$, where *m* ∈ [1, 2, …, $3^{n}$]		
	**L2**	**For each** hyperparameter set $a_{i}{}^{r},\;\mathrm{where}\;i$ ∈ [1, 2, …, *n*] (i=0 intially)	
			**If** $P_{m}{}^{r}$ (performance of model by using hyperparameters $a_{i}{}^{r}\left[0\right]$ ($a_{i}{}^{r}\left[0\right]=k_{i})$, $a_{i+1}{}^{r}$, …, $a_{n}{}^{r}$) < $P_{m+1}{}^{r}\;$(performance of model by using hyperparameters $a_{i}{}^{0}\left[1\right]$ $(a_{i}{}^{r}\left[1\right]=2\times k_{i}$), $a_{i+1}{}^{r}$, …, $a_{n}{}^{r}$) < $P_{m+2}{}^{r}$ (performance of model by using hyperparameters $a_{i}{}^{r}\left[2\right]$ $(a_{i}{}^{r}\left[2\right]$ = 8 $\times k_{i})$, $a_{i+1}{}^{r}$, …, $a_{n}{}^{r}$) **do** Generate a new set for $a_{i}{}^{r+1}$ that $a_{i}{}^{r+1}\in \left[a_{i}{}^{r}\left[2\right],2\times a_{i}{}^{r}\left[2\right],8\times a_{i}{}^{r}\left[2\right]\right]$
			**Else if** $P_{m}{}^{r}$ < $P_{m+1}{}^{r}\mathrm{and}$ $P_{m+1}{}^{r}>P_{m+2}{}^{r}\;$**do** Generate a new set for $a_{i}{}^{r+1}$ that $a_{i}{}^{r+1}\in \left[\frac{\left(\;a_{i}{}^{r}\left[0\right]+a_{i}{}^{r}\left[1\right]\right)}{2},\;a_{i}{}^{r}\left[1\right],\frac{\left(\;a_{i}{}^{r}\left[1\right]+a_{i}{}^{r}\left[2\right]\right)}{2}\right]$
			**Else do** Generate a new set for $a_{i}{}^{r+1}$ that $a_{i}{}^{r+1}\in \left[\frac{1}{8}\times a_{i}{}^{r}\left[0\right],\;\frac{1}{2}\times a_{i}{}^{r}\left[0\right],a_{i}{}^{r}\left[0\right]\right]$ **End if**
			**i = i + 1, Repeat L2**
	**r = r + 1, Repeat L1**		
		**If** $(P_{i}{}^{b}$ < $P_{i+1}{}^{b}$ < $P_{i+2}{}^{b}$ **and**$\;P_{i}{}^{b+1}$ > $P_{i+1}{}^{b+1}>P_{i+2}{}^{b+1})$ **or** ($P_{i}{}^{b}$ > $P_{i+1}{}^{b}>P_{i+2}{}^{b}\;\mathrm{and}\;P_{i}{}^{b+1}$ < $P_{i+1}{}^{b+1}$ < $P_{i+2}{}^{b+1}$) **do** Generate a new set for $a_{i}{}^{b+1}$ that $a_{i}{}^{b+1}\in \left[\frac{a_{i}{}^{b}\left[0\right]+a_{i}{}^{b}\left[1\right]}{2},a_{i}{}^{b}\left[1\right],\frac{a_{i}{}^{b}\left[1\right]+a_{i}{}^{b}\left[2\right]}{2}\right]$ **End if**	
	**End L1 For**		

**Table 2:** The chosen hyperparameter sets for the first round of AGD.

Hyperparameter Set ($a_{i}{}^{0}$)	Lower Value ($a_{i}{}^{0}\left[0\right]$)	Mid Value ($a_{i}{}^{0}\left[1\right]$)	Higher Value ($a_{i}{}^{0}\left[2\right]$)
$N_{k1}$	$N_{ch}$	$2\times N_{ch}$	$8\times N_{ch}$
$N_{k2}$	$N_{ch}$	$2\times N_{ch}$	$8\times N_{ch}$
$K_{2}$	$N_{ch}$	$2\times N_{ch}$	$8\times N_{ch}$
$S_{2}$	$\frac{1}{8}\times K_{2}$	$\frac{1}{4}\times K_{2}$	$K_{2}$
$N_{fb}$	1	3	7

**Table 3:** Hyperparameter sets for the second round of AGD.

Hyperparameter Set	Hyperparameters	Lower Bound	Mid Value	Higher Bound
Set_A (AGD round 2)	$K_{2\;}$$(\mathrm{Maximum}\;\mathrm{value}\;\mathrm{of}\;K_{2}\;is\;2\times N_{FFT}\times N_{fb}$)	$\mathrm{Argmax}\{8\times N_{ch},\;2\times N_{FFT}\times N_{fb}\}$	$\mathrm{Argmax}\{16\times N_{ch},\;2\times N_{FFT}\times N_{fb}\}$	Argmax$\left\{64\times N_{ch},\;2\times N_{FFT}\times N_{fb}\right\}$
	$S_{2}$	$\frac{1}{64}\times K_{2}$	$\frac{1}{32}\times K_{2}$	$\frac{1}{8}\times K_{2}$
Set_B (AGD round 2)	$K_{2}$$(\mathrm{Maximum}\;\mathrm{value}\;\mathrm{of}\;K_{2}\;is\;2\times N_{FFT}\times N_{fb}$)	$\mathrm{Argmax}\{8\times N_{ch},\;2\times N_{FFT}\times N_{fb}\}$	$\mathrm{Argmax}\{16\times N_{ch},\;2\times N_{FFT}\times N_{fb}\}$	Argmax$\left\{64\times N_{ch},\;2\times N_{FFT}\times N_{fb}\right\}$
	$S_{2}$	$K_{2}$	$K_{2}$	$K_{2}$

**Table 4:** Optimized hyperparameter set after the second round of AGD.

Hyperparameter	One Filter Bank Hyperparameter Set		Three Filter Banks Hyperparameter Set	
	Channel Number-Based Representation	Fixed Value Representation	Channel Number-Based Representation	Fixed Value Representation
$N_{fb}$	1	1	3	3
$N_{k1}$	$8\times N_{ch}$	64	$8\times N_{ch}$	64
$N_{k2}$	$2\times N_{ch}$	16	$8\times N_{ch}$	64
$K_{2}$	$8\times N_{ch}$	64	$8\times N_{ch}$	64`
$S_{2}$	$N_{ch}$	8	$8\times N_{ch}$	64

**Table 5:** Hyperparameters of FB-CCNN using the Nakanishi dataset and Benchmark dataset. For the Nakanishi dataset, the fixed value hyperparameter set is the same as the channel number-based hyperparameter set.

Hyper Parameters	Fixed Value Hyperparameter Set		Channel Number-Based Hyperparameter Set			
			Nakanishi Dataset		Benchmark Dataset	
$N_{ch}$	8, 64	8, 64	8		64	
$N_{fb}$	1	3	1	3	1	3
$N_{k1}$	64	64	$8\times N_{ch}$(64)	$8\times N_{ch}$(64)	$8\times N_{ch}$ (512)	$8\times N_{ch}$ (512)
$N_{k2}$	16	64	$2\times N_{ch}$(16)	$8\times N_{ch}$(64)	$2\times N_{ch}$ (128)	$8\times N_{ch}$ (512)
$K_{2}$	64	64	$8\times N_{ch}$(64)	$8\times N_{ch}$(64)	$8\times N_{ch}$ (512)	$8\times N_{ch}$ (512)
$S_{2}$	8	64	$N_{ch}$(8)	$8\times N_{ch}$(64)	$N_{ch}$(64)	$8\times N_{ch}$ (512)

**Table 6:** Performances of FB-CCNN and benchmark methods using the Nakanishi dataset.

Subject	CCA	C-CNN	FB-CNN	FB-CCNN (FB = 1)	FB-CCNN (FB = 3)
S1	29.17	75.69	91.67	87.90	90.05
S2	26.25	51.81	57.08	78.58	80.02
S3	59.44	93.89	97.36	93.37	92.38
S4	80.28	98.61	98.11	95.88	95.97
S5	52.36	99.72	99.58	99.73	99.37
S6	87.22	99.72	99.95	99.46	99.55
S7	69.17	92.64	98.75	98.66	99.55
S8	96.67	99.03	99.58	99.19	99.19
S9	66.39	97.36	97.92	98.03	98.03
S10	65.28	90.28	91.94	92.38	94.35
Average	63.22 ± 22.84	89.88 ± 15.22	93.19 ± 13.04	94.32 ± 6.73	**94.85 ± 6.18**

**Table 7:** Performances of FB-CCNN and benchmark methods using the Benchmark dataset. FB represents the filter bank number, and ch indicates that the hyperparameter set is channel number based.

Subject	M-CNN	C-CNN	FB-CNN	FB-CCNN (FB = 1)	FB-CCNN (FB = 3)	FB-CCNN (FB = 1, ch)	FB-CCNN (FB = 3, ch)
S1	71.33	73.58	85.42	80.25	81.12	74.31	82.50
S2	78.00	88.58	92.92	86.19	85.06	78.94	85.13
S3	84.67	89.25	93.08	84.88	86.81	74.31	74.31
S4	83.67	90.50	94.17	96.25	97.69	83.50	83.50
S5	79.92	87.00	93.17	86.31	90.00	66.69	81.63
S6	70.16	80.16	83.75	67.13	76.63	63.50	76.75
S7	50.75	73.88	75.25	73.56	76.50	51.69	64.31
S8	52.67	61.66	67.00	56.69	72.94	48.56	54.38
S9	61.25	68.25	71.25	74.44	76.31	69.56	72.56
S10	69.47	78.58	91.25	90.00	94.31	70.63	84.63
S11	34.83	38.75	43.67	61.87	53.88	49.88	48.38
S12	83.16	86.92	86.75	72.63	88.69	73.94	84.44
S13	64.67	72.42	82.83	80.81	74.13	66.88	51.00
S14	78.67	81.08	84.92	96.06	96.56	63.13	91.88
S15	51.92	64.42	61.32	58.25	74.00	54.50	63.00
S16	56.00	72.00	77.25	79.69	84.56	70.50	84.19
S17	54.19	70.91	72.75	70.31	75.31	67.50	43.50
S18	51.75	62.08	65.50	35.63	57.38	43.31	58.63
S19	33.67	36.41	43.67	80.13	81.31	72.75	79.75
S20	69.33	78.00	87.25	85.88	85.69	72.38	75.88
S21	78.38	85.42	86.50	74.13	77.50	71.00	67.19
S22	88.33	91.42	94.67	93.56	96.69	88.13	92.81
S23	74.67	77.83	83.92	87.00	87.13	69.63	66.50
S24	78.29	81.92	85.25	81.38	82.31	67.81	79.44
S25	76.75	80.00	81.33	57.19	69.69	47.88	56.88
S26	82.25	84.92	85.17	73.00	83.56	54.88	65.63
S27	88.16	94.16	94.25	86.13	90.50	78.75	86.06
S28	73.16	84.08	90.50	85.06	92.19	68.44	88.69
S29	32.41	46.75	49.50	43.88	50.25	20.88	26.06
S30	67.67	83.00	81.50	78.69	83.81	72.19	75.19
S31	87.13	96.50	96.58	72.75	97.44	67.50	88.56
S32	91.92	94.00	95.92	99.25	98.56	95.13	99.25
S33	22.58	28.72	36.25	27.50	32.13	10.81	13.56
S34	64.48	76.75	76.00	80.50	82.06	69.31	75.06
S35	74.58	74.08	76.17	84.44	87.50	73.31	83.25
Average	67.45 ± 17.62	75.26 ± 16.43	79.05 ± 15.86	75.47 ± 16.40	**80.58 ± 14.43**	64.92 ± 16.62	71.56 ± 18.69

## Data Availability

All of the data and code used in this article can be accessed at: https://drive.google.com/drive/folders/18d-txkXeLuuvMU7rBbjjD7fnqbweAK_-?usp=sharing (accessed on 14 April 2023).
